# Shame on Me? Love Me Tender! Inducing and Reducing Shame and Fear in Social Anxiety in an Analogous Sample

**DOI:** 10.32872/cpe.7895

**Published:** 2023-09-29

**Authors:** Jakob Fink-Lamotte, Jürgen Hoyer, Pauline Platter, Christian Stierle, Cornelia Exner

**Affiliations:** 1 Clinical Psychology, University of Potsdam, Potsdam, Germany; 2 Clinical Psychology and Psychotherapy, University of Leipzig, Leipzig, Germany; 3 Clinical Psychology and Psychotherapy, Technische Universität Dresden, Dresden, Germany; 4 Hochschule Fresenius für Wirtschaft und Medien, Hamburg, Germany; Philipps-University of Marburg, Marburg, Germany

**Keywords:** social anxiety, shame, shame induction, self-compassion, reappraisal

## Abstract

**Background:**

Shame is considered an important factor in the development and maintenance of many psychological disorders, e.g., social anxiety disorder, and an interesting target point for therapeutic intervention.

**Method:**

In the present experimental study, we used an online-adopted Autobiographical Emotional Memory Task (AEMT) to induce shame and tested different micro-interventions (self-compassion, cognitive reappraisal, and a control intervention) with respect to their potential to reduce shame intensity. One-hundred-and-fifteen healthy subjects participated in the study and completed a series of self-report questionnaires on self-compassion, shame, and social anxiety.

**Results:**

The experimental shame induction was well accepted and successful (with significantly heightened feelings of shame); there were no study drop-outs. There was a significant time*condition interaction, which was due the self-compassion-based intervention resulting in a significantly larger reduction of shame than the control condition (counting fishes). In addition, the main effect of the factor experimental condition was further moderated (enhanced) by trait social anxiety and trait self-compassion.

**Conclusion:**

The findings demonstrate the usefulness of online-adopted AEMT for the experimental induction of shame. They suggest that especially self-compassion interventions can be beneficial in alleviating intense shame experiences, which is in accordance with self-compassion theory. Overall, the results are promising in the context of experimental shame research and its potential clinical impacts call for further replication.

## Highlights

- We targeted shame by testing micro-interventions to reduce shame intensity.
- Shame was successfully induced using an online Autobiographical Emotional Memory Task in an experimental design.
- Self-compassion is a significant intervention to reduce shame.
- The results have promising clinical implications as well as for future research.

Shame can broadly be understood as a global devaluation of the self and is characterized by a critical, judgmental, and condemning self-verbalization (self-directed private speech; (Lewis, 1971). As shame motivates people to view themselves critically, they behave in a more reserved and detached manner in social situations. Fessler (2004) argues that the psychological function of this behavior (as a “defense mechanism”) might be to protect us from the rejection of others. As a state, shame feels like being unmasked, judged, and humiliated in a specific situation (Tangney et al., 2005), while as a trait shame comprises the tendency to experience these feelings in a variety of different (social) situations.

When a strong desire for positive reactions from others is combined with a high level of insecurity about it, people might feel exaggerated shame (Schuster et al., 2021). This ambivalence of desire for recognition and interactional insecurity leads to constant self-critical monitoring, which can be an underlying mechanism of psychological disorders. Shame associated excessive self-attention and adopting an observer perspective (self-as-object) are central factors of Clark and Wells (1995) psychopathological model of social anxiety disorder (SAD). Subsequently, exaggerated shame is thought to be a particularly important maintaining factor for SAD (Gilbert & Miles, 2000; Hedman et al., 2013), although it further plays a crucial role in the development and maintenance of a variety of psychopathological disorders e.g. depression (for review: Kim et al., 2011), eating disorders (Nechita et al., 2021), post-traumatic stress disorder (Saraiya & Lopez-Castro, 2016). In order to avoid experiencing such shame and the rejection of others, people who suffer from these disorders avoid social situations to varying extents.

SAD is not only a highly prevalent but also a highly debilitating disorder (Fehm et al., 2005; Kessler, 2003). Several studies show a significant positive correlation between shame proneness and SAD (Fergus et al., 2010; Gilbert & Miles, 2000; Hedman et al., 2013; Schuster et al., 2021; Swee et al., 2021), although research on interventions specifically focusing on reducing (or preventing) exaggerated shame in SAD is scarce. Furthermore, there is a lack of experimental studies on the modification of shame to isolate theoretically important change processes. This is supported by a review of Goffnett et al. (2020), which only includes one study investigating interventions to change shame in the context of SAD.

Nonetheless, the review also showed the promising effect of psychotherapeutic interventions with a significant reduction in shame in a post-test in 89% across a variety of contextual aspects (PTSD, body image, borderline personality disorder, etc.). Most of these studies used interventions based on cognitive-behavioral therapy (CBT) and mindfulness, while four of them applied compassion-focused interventions. Compassion-focused therapy (CFT; Gilbert, 2010) is not only a promising approach for treating SAD, for example (Blackie & Kocovski, 2018; Goldin & Gross, 2010; Koszycki et al., 2016). Self-compassion is a central construct of CFT, which can be understood as a friendly and understanding self-perspective in difficult situations characterized by an understanding that suffering is an inevitable part of human nature, while accepting it in a mindful manner (Neff, 2003b). Nonetheless, self-compassion is more than simply friendliness; rather, it is about awareness of pain that may be present and having the intention to try to alleviate it (Gilbert, 2010). Studies have shown that patients with SAD have lower self-compassion than healthy individuals (Werner et al., 2012) and an intervention based on self-compassion can effectively reduce shame (see review of Goffnett et al. (2020). This preliminary evidence suggests that CFT might be especially efficacious for the treatment of exaggerated shame, quite in accordance with the underlying theory: whereas shame is associated with a global negative devaluation of the self, self-compassion clearly counteracts this tendency as it promotes a loving relationship with the self. While shame involves a severe and judgmental emotional relationship with the self, self-compassion teaches an empathic approach.

However, as common factors in psychotherapy might mask the effects of specific interventions, head-to-head comparison studies disentangling the most effective components of psychotherapy can only be successful when based on extremely large patient samples. As Mulder et al. (2017) suggest, studies based on online-based interventions that aim at transdiagnostic processes (such as shame) are very promising. Thus, it is hypothesized that process-level variance can be more accurately elucidated by holding therapist variance constant. Further, Hofmann and Hayes (2019) suggested a paradigm switch to a process-based therapy approach where moderators and mediators of clinical change are at the center of clinical research. We would like to add the notion that experimental studies that use micro-interventions and isolate theoretically important change processes like *trait social anxiety*, *trait self****-****compassion* and *trait shame* could also help to transcend the common factors problem (*Do psychotherapies work primarily through the specific factors described in treatment manuals, or common factors such as therapeutic relationship, expectations, confronting problems, mastery, and attribution of the outcome*?).

To test the specific effectiveness of CFT for shame in the context of different levels of social anxiety symptoms, we compared its effects with those of another established evidence-based emotion-regulation condition, cognitive reappraisal (REAP), which is one of the best-evaluated emotion-regulation strategies (Ochsner & Gross, 2007). Gross and Thompson (2007) defined reappraisal as changing “a situation’s meaning in a way that alters its emotional impact” (p. 20). There is evidence that reappraisal can be helpful in reducing symptoms of social anxiety (for a review, see Dryman & Heimberg, 2018). This study thus aimed to test whether a self-compassion micro-intervention (COMP) is superior in reducing shame in subjects with different levels of SA symptoms compared to a REAP intervention and a control micro-intervention (CONT).

In the present study, the *Autobiographical Emotional Memory Task* (AEMT) (Mills & D’Mello, 2014; Prkachin et al., 1999) was used, as a method that has been proven to successfully induce shame (de Hooge et al., 2010; Friis et al., 2017; Houazene et al., 2021; Keng & Tan, 2017). In the AEMT, participants are instructed to remember a recent embarrassing social situation and focus on the associated emotions and feelings associated. To induce shame in an online experiment, we modified the AEMT by including more detailed audio instructions. Therefore, a further aim of the present study was to first generate data on the validity of the online version of the AEMT and subsequently to test for the shame-specificity of the AEMT. We define the manipulation check as successful when a) state shame is efficiently induced in all three micro-intervention conditions and b) the increase of state shame is more pronounced compared to state fear.

Due to theoretical assumptions on the specific effects of self-compassion for shame, we expect that COMP will reduce shame more effectively than the REAP and the CONT (H 1). We also expect that the experimental induction of shame should lead to a higher level of shame in subjects with higher rather than lower levels of social anxiety (H 2.1). We thus expect that REAP and COMP result in a stronger reduction of shame and fear compared to CONT in subjects with lower compared to higher levels of social anxiety (H 2.2: interaction of condition and anxiety group).

## Material and Method

### Participants

The participants were recruited using a University of Leipzig internal database. As compensation for their participation, they either took part in a lottery (five vouchers worth 10 €) or received course credit. One-hundred-and-forty-four non-clinical subjects volunteered to participate and all provided written informed consent. Respondents had to actively tick whether the following inclusion and exclusion criteria applied.

Inclusion criteria: good language skills in German, aged between 18 and 65 years, being in a quite environment and having the ability to listen to audio files.

Exclusion criteria: being pregnant, suffering from a severe mental disorder other than social phobia or a severe health impairment, or a neurological disease (e.g. traumatic brain injury, falls with unconsciousness, neurodegenerative diseases, strokes, tic disorders), psychotropic substance abuse (except coffee and nicotine) or benzodiazepine or neuroleptic medication.

Subjects could not continue the experiment if they denied presence of one of the inclusion criteria or agreed with the presence of one of the exclusion criteria

At the end of the experiment, all subjects were asked if “something unusual” happened during the experiment. Based on the responses, *n* = 12 subjects were excluded due to self-reported distraction, *n* = 2 were excluded due to self-reported technical problems, *n* = 2 due to unreasonably long experiment durations, *n* = 3 because of a more than 2 *SD* variance in trait questionnaires and *n* = 10 subjects were excluded because they stated that they had not carried out the micro-interventions at all, had dropped out beforehand, or had not mentioned anything at all concerning the interventions whereby it was not ensured that these subjects heard the intervention at all. In total, one-hundred-and-fifteen subjects were included in the statistical analysis, of whom *n* = 39 had been randomly assigned to COMP, *n* = 37 to REAP, and *n* = 39 to the CONT.

### Measures

#### Trait Shame: Tangney´s Test of Self-Conscious Affect

The level of shame proneness was assessed using the German version (Rüsch et al., 2007) of *Tangney´s Test of Self-Conscious Affect* (TOSCA-3; Tangney et al., 2000). The TOSCA-3, presenting 11 scenes (“You have broken an object at work and then hide it.”) with four reactions (e.g. “You would think about resigning.”) rated from 1, “not likely", to 5, “very likely", has been reported to have a high internal consistency (Cronbach’s α > .77, in this study α = .66). The TOSCA-3 results in sum-scores for shame-proneness between 11 (low shame proneness) and 55 (high shame proneness).

#### Social Anxiety: Social Interaction Anxiety Scale

The severity of social anxiety was assessed using the German version (Stangier et al., 1999) of the *Social Interaction Anxiety Scale* (SIAS; Mattick & Clarke, 1998). The 20-item scale (e.g. “I have difficulty making eye contact with others”), rated from 0, “not applicable at all", to 4, “very much applicable", has a high internal consistency (patients with SAD; *N* = 66; α = .86; healthy controls; *N* = 50; α = .90, in this study α = .94). The sum-scores ranging from 0 to 80. In the study of Stangier et al. (1999) the social anxiety group showed a mean sum-score of 40.8 (*SD* = 16.6), the non-clinical group showed a mean sum-score of 12.5 (*SD* = 5.7).

#### Trait Self-Compassion: Self-Compassion Scale

The *Self-Compassion Scale* (SCS; Neff, 2003a; German version SCS-D; Hupfeld & Ruffieux, 2011) was applied to measure the trait of self-compassion. The 26-item scale (e.g. “I disapprove and condemn my own faults and weaknesses.”), rated from 1, “almost never", to 5, “almost always", has been reported to have a high internal consistency (Cronbach’s α = .91, in this study α = .89). The SCS results in mean-scores for self-compassion trait between 1 (low) and 5 (high).

#### State-Trait Anxiety Inventory for State Anxiety

The short version of the *State-Trait Anxiety Inventory for State Anxiety* (STAI-SKD; Englert et al., 2011) was applied to measure the level of state fear. The 5-item German translation (e.g. “I am nervous”), rated from 1, “not at all", to 5, “very much", has a high internal consistency (Cronbach’s α > .84, in this study α = .9). The STAI-SKD results in mean-scores for state anxiety between 1 (low) and 5 (high).

#### State Shame and Guilt Scale

The state variable shame was assessed in questionnaire format using self-assessment via the five shame items (e.g. “I feel small and insignificant”), rated from 0, “not applicable at all", to 4, “very much applicable", of the *State Shame and Guilt Scale* (SSGS; Marschall et al., 1994). An example item for shame is “I want to sink into the ground and disappear.” The SSGS results in mean-scores for state shame between 1 (low) and 5 (high) and has a high internal consistency (this study: Cronbach’s α = .93).

### Experimental Design

The influence of self-compassion vs. cognitive reappraisal vs. control on shame and fear was tested in an online experiment using Unipark with a mixed subject design. While the differences between the COMP, REAP, and CONT were analyzed by a between-subject design, time was assessed in a within-subject design. Therefore, three data points (baseline, t0; post-induction, t1; post-intervention, t2) were recorded for each participant. In the study documentation, we reported how we determined our sample size, all data exclusion (if any), and all manipulations and measures conducted.

### Procedure

**Figure 1:**
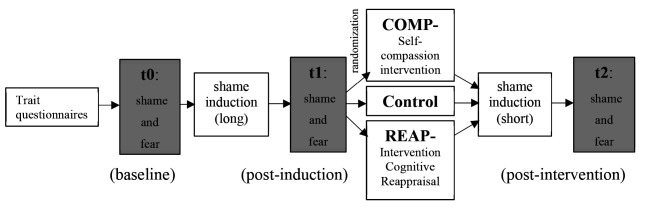
Illustration of the Experimental Procedure

The data was collected between June and November 2020. On average, one trial lasted 42.3 minutes (*SD* = 16.14 minutes) and all experimental conditions took the same time, *F*(2,109) = .104, *p* = .901, *d* = .002. The participants were first informed of the details of the study and the test subjects’ written consent to participate was obtained. The participants then had to fill out the questionnaires listed above and state fear and shame were assessed with SSGS and STAI-SKD (t0). Thereafter, shame was induced using auditory instructions transmitted via headphones (see section “Shame Induction”). In the next step, state fear and shame were assessed again (t1) before participants received their randomization result and performed one of the three experimental conditions (COMP vs. REAP vs. CONT, see sections “Experimental Conditions”). Audio instructions were given over headphones. Then, shame was induced again, followed by the third assessment of state fear and shame (t2). Finally, questions regarding the usability and effectiveness of the manipulation ended the trial (see section “Manipulation Check”), followed by a debriefing. The schema of the experimental trial is shown in Figure 1.

#### Shame Induction

Shame was induced using an auditive *Autobiographical Emotional Memory Task* (Mills & D’Mello, 2014; Prkachin et al., 1999). The participants were instructed to remember a humiliating social situation and to focus on the emotions and feelings associated with it. If they could not think of such a situation, they were given another auditive instruction (ICD-10 SAD diagnostic criteria: e.g., focusing attention towards oneself, feeling physiological exacerbation). Overall, the manipulation took six minutes. To isolate the intervention’s core effect from mere time-effects, all participants received a 3-minute short version of the AEMT after the intervention for reasons of comparison (Appendix A, Supplementary Materials).

#### Experimental Condition: Self-Compassion Intervention (COMP)

In the self-compassion intervention (COMP), the subjects received auditory training to enhance self-compassion (Desmond, 2017; Gilbert, 2013; Neff, 2003b). In different sections, the participants were guided through an imagination exercise to increase mindfulness and acceptance, to feel human connectedness, and to build self-friendliness and wisdom. The training was adapted from a previously used intervention by Fink-Lamotte et al. (2022) to the context of social anxiety and the duration of the procedure was 8 minutes in total. A transcript of the instructions is attached in Appendix B, Supplementary Materials.

#### Experimental Condition: Cognitive Reappraisal Intervention (REAP)

In the cognitive reappraisal intervention (REAP), the subjects received an audio instruction to reevaluate maladaptive cognitions in social situations based on a previously used intervention by Fink et al. (2018). In the context of a guided imagination exercise, the intervention aimed to reflect on factual knowledge, decatastrophize, and strengthen self-efficacy as well as appraise an alternative and more positive and empowering perception of the social situation. The duration of the procedure was 6:49 minutes in total and a transcript of the instructions is attached in Appendix C, Supplementary Materials.

#### Control Condition: Counting Fishes (CONT)

In the control condition, the participants had to watch a video of an aquarium with moving fishes. They were instructed to count the number of times a yellow fish swam in and out of the picture. The duration of the procedure was 6:30 minutes in total and the experiment was adapted from Fink et al. (2018) and Fink and Exner (2019).

#### Manipulation Check

To check if the manipulation induced shame and/or anxiety, the participants received a four-item questionnaire. Similarly, four items assessed the subjective evaluation of the effectiveness of the instructions provided. In addition, we invited participants to describe their personal experiences and strategies during the intervention (see Appendix D, Supplementary Materials, for all materials concerning the manipulation-check). After the intervention, the participants were also asked to name specific aspects of the intervention that they perceived as helpful or hindering (Appendix E, Supplementary Materials).

### Statistical Analysis

The software R (R Development Core Team, 2020) and JASP (JASP Team, 2020) were used for the statistical analysis. The statistical investigations were tested at the α = .05 (two-tailed) level of significance. To test the manipulation check, two repeated ANOVAs investigating the effects of *condition* (COMP/REAP/CONT) and *anxiety group*, dividing the sample by a median split into low and high socially anxious groups, between t0 (before induction) and t1 (after induction), with t0 as a covariate, were calculated. These ANOVAs were run for both dependent variables *shame and fear*, specifically testing the within-subject factor *time* for induction (t0/t1) and intervention (t1/t2), and were followed by Bonferroni-corrected post-hoc tests for significant main effects and interactions. We are aware, that the Media-Split increases the probability of type I errors (Maxwell & Delaney, 1993), so we additionally ran two General Linear Mixed Model (GLMM), with the continuous variable Trait Anxiety (sensitive analysis), which led to comparable results.

Furthermore, two ANCOVAs investigating the effects of *condition* and *anxiety group* for the difference t1 (after induction) – t2 (after intervention) as dependent variable for shame and fear, with t1 as a covariate, were calculated. The addition of t1 (t0) as a covariate is to ensure that any change observed is not artifactually due to high t1 (t0) values (regression to the mean). If the effect of the condition is significant, the ANCOVA was repeated for the pairs of conditions (each intervention is compared separately with the control) to test whether a difference between the interventions is greater than chance as indicated by the change in the control condition, which in turn would be tested by the condition term in the ANCOVA.

An overall ANOVA was not calculated, because we expected an independent induction and an independent intervention effect. The effect sizes were calculated using the R package “rstatix” (Version 0.4.0; Kassambara, 2019) whereby the adjusted partial eta-squares ($\eta_{p}^{2}$) are reported. A Shapiro Wilk Test for normality was conducted, and the normality assumption was violated for both dependent variables. However, due to the sample size, it is possible to assume an approximate asymptotic normal distribution for each of these variables (Field, 2013). A Levene test for the homogeneity of variance was conducted for the dependent variables across the time and the homogeneity of variance was not violated for shame and fear at any time point, *p* > .05. To exploratively test the effect of individual traits on shame and fear reduction, either an analysis of covariance (ANCOVA) or correlational analyses (Pearson’s product-moment correlations) was calculated, depending on whether the experimental conditions differed or not.

## Results

### Demographic Characteristics

The three conditions were not statistically different concerning age, sex, level of social anxiety (SIAS), level of shame (TOSCA-3), level of self-compassion, or any of the other demographic or clinical data (see Table 1). The median-split resulted in a low socially anxious group (*n* = 56, sex: 51 females [91%], age = 29.86 [*SD* = 11.54]) and a high socially anxious group (*n* = 59, sex: 45 females [76%], age = 28.98 [*SD* = 8.77]). These two groups did not differ a priori concerning age, *t*(113) = .456, *p* = .647, *d* = .086, trait compassion, *t*(113) = .784, *p* = .435, *d* = .146, and trait shame, *t*(113) = .292, *p* = .771, *d* = .055, but did differ – as expected – concerning social anxiousness, *t*(113) = .14.01, *p* < .001, *d* = 2.61.

**Table 1:** Demographics and Clinical Characteristics by Condition

Characteristics	COMP (*n* = 39)		REAP (*n* = 37)		CONT (*n* = 39)		stats	*p*	$\eta_{p}^{2}$
	*M*	*SD* / %	*M*	*SD* / %	*M*	*SD* / %			
Sex (% female)	33:6	86%	31:6	84%	32:7	82%	X^2^(2) = .064	.969	
Age	28.49	8.019	31.76	12.23	28.10	9.935	*F*(2, 112) = 1.810	.168	.032
Highest Education^a^	2.821	.644	2.757	.641	2.872	.409	*F*(2, 112) = 0.382	.683	.007
Social Anxiety	33.05	21.15	28.30	16.69	26.23	14.24	*F*(2, 112) = 1.535	.220	.027
Shame	33.63	2.56	32.22	2.729	32.68	2.85	*F*(2, 112) = 2.674	.073	.046
Compassion^b^	3.29	.32	3.17	.38	3.23	.38	*F*(2, 112) = 1.036	.358	.018

*Note.* COMP = participants with the self-compassion intervention; REAP = participants with the cognitive reappraisal intervention; Social Anxiety (SIAS = Social-Interaction-Anxiety-Scale); Shame (TOSCA-3 = Tangney’s Test of Self-Conscious Affect) Compassion (SCS = Self-Compassion Scale).

^a^Educational level was recorded in four levels matching the German school system from 1 [= highest secondary school level achieved (Abitur)] to 4 [= basic secondary school level achieved (Hauptschule)]. ^b^Self-compassion level was the average of the SCS-Score without the self-criticism subscales (Neff, 2003a).

### Hypothesis Testing

#### Manipulation Check: Induction Between t0 and t1

**Figure 2:**
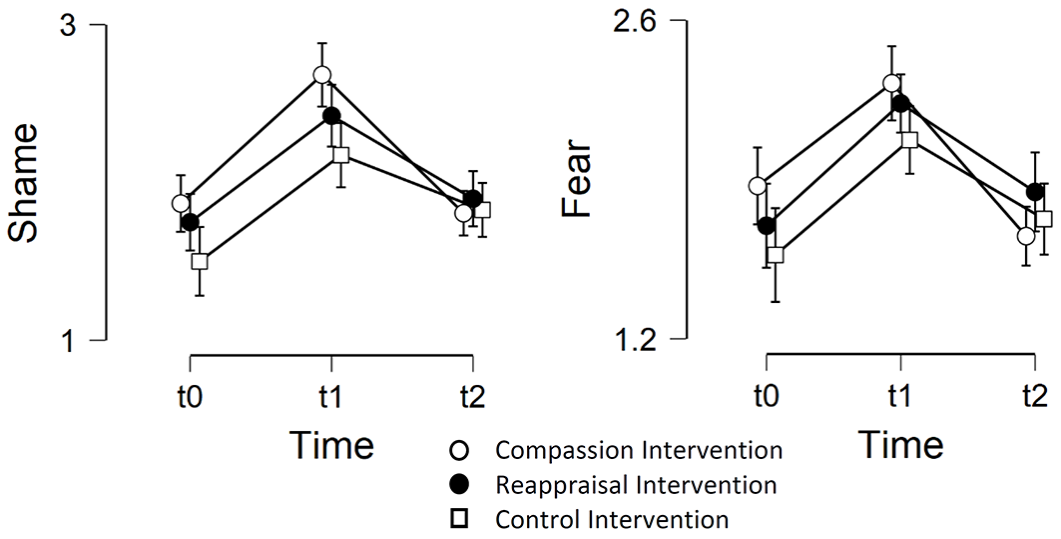
Means and Standard Error Bars of the Shame and Fear Experience Between t0 (Baseline), t1 (Post-Induction), and t2 (Post-Intervention) Across the Three Experimental Conditions *Note.* Shame ratings (a) and fear ratings (b) were given on a scale between 1 and 4.

A repeated-measured ANOVA investigating the effects of *condition* and *anxiety group* between t0 (before induction) and t1 (after induction) for shame as dependent variable, with t0 as a covariate, shows a significant main effect of the *covariate t0*, *F*(1,108) = 116.935, *p* < .001, $\eta_{p}^{2}$ = .73, a main significant effect of *time*, *F*(1,108) = 47.233, *p* < .001, $\eta_{p}^{2}$ = .304, and a significant main effect of *anxiety group*, *F*(1,108) = 4.998, *p* = .027, $\eta_{p}^{2}$ = .044, but no significant main effect for *condition*, nor any other significant interaction, *p* > .37, $\eta_{p}^{2}$ < .01. The Bonferroni-corrected post-hoc results, controlled for the covariate, show a stronger shame experience in t1 compared to t0, *M* = .767, *t* = 8.952, *p* < .001, *d* = .835. However according to the Bonferroni-corrected post-hoc results, there was no significant stronger shame experience in the high socially anxious group, *M* = .09, *t* = -.859, *p* = .392, *d* = .08, compared to the low socially anxious group (see Figure 2a).

A repeated-measured ANOVA investigating the effects of *condition* and *anxiety group* between t0 (before induction) and t1 (after induction) for fear as dependent variable, with t0 as a covariate, shows a main significant effect of the *covariate t0*, *F*(1,108) = 291.32, *p* < .001, $\eta_{p}^{2}$ = .73, a main significant effect of *time*, *F*(1,108) = 18.607, *p* < .001, $\eta_{p}^{2}$ = .147, and a significant main effect of *anxiety group*, *F*(1,108) = 4.44, *p* = .037, $\eta_{p}^{2}$ = .039, but no significant main effect for *condition*, nor any other significant interaction, *p* > .41, $\eta_{p}^{2}$ < .02. The Bonferroni-corrected post-hoc results, controlled for the covariate, show a stronger fear experience in t1 compared to t0, *M* = .51, *t* = 7.247, *p* < .001, *d* = .676 as well as a stronger fear experience in the high socially anxious group, *M* = .15, *t* = -2.39, *p* = .019, *d* = .22, compared to the low socially anxious group (see Figure 2b).

#### Hypothesis 1 and 2: Emotion Regulation Between t1 and t2 and the Impact of Group

An ANCOVA investigating the effects of *condition* and *anxiety group* for the difference t1 (after induction) – t2 (after intervention) as dependent variable for shame, with t1 as a covariate, shows a significant main effect of the *covariate t1*, *F*(1,108) = 43.323, *p* < .001, $\eta_{p}^{2}$ = .267, a marginally significant main effect of *condition*, *F*(2,108) = 2.98, *p* = .055, $\eta_{p}^{2}$ = .037, and a significant main effect of *anxiety group*, *F*(1,108) = 4.378, *p* = .039, $\eta_{p}^{2}$ = .027, but no significant interaction effect of *condition* and *anxiety group, p* > .85, $\eta_{p}^{2}$ < .01. The Tukey-corrected post-hoc results, controlled for the covariate, show a stronger shame reduction in the COMP compared to the CONT condition, *M* = .395, *t* = -2.441, *p* = .043, *d* = .523, but no significant differences between COMP and REAP, *M* = .204, *t* = -1.253, *p* = .425, *d* = .24, and REAP and COMP, *M* = .191, *t* = -1.175, *p* = .471, *d* = .224, as well as a stronger shame reduction in the high socially anxious group, *M* = .311, *t* = -2.092, *p* = .039, *d* = .37, compared to the low socially anxious group. The other post-hoc comparisons became not significant, *p* > .42, *d* < .22 (see Figure 2a).

An ANCOVA investigating the effects of *condition* and *anxiety group* for the difference t1 (after induction) – t2 (after intervention) as dependent variable for fear, with t1 as a covariate, shows a significant main effect of the *covariate t1*, *F*(1,108) = 28.24, *p* < .001, $\eta_{p}^{2}$ = .19, but no significant main effects of *condition* and *anxiety group,* nor a significant interaction effect, *p* > .42, *d* < .22 (see Figure 2b).

### Explorative Analysis: Effects of Individual Traits on Changing Shame

#### Effect of Individual Traits on Shame Induction

While the factor *anxiety group* was unrelated to shame induction, we also calculated Pearson’s product-moment correlations between the dependent variable *shame induction between t0 and t1* and the individual traits. The correlations between *trait social anxiety*, *trait self-compassion*, *trait shame* and the shame induction were all insignificant (all *r* between .07 and -.07, all *p* > .45).

#### Effect of Individual Traits on Shame Reduction

To examine the association between the trait variables and shame *reduction* in more detail, an ANCOVA investigating the effect of the three *conditions* for the difference t1 – t2 for shame, with t1, *trait social anxiety, trait self-compassion* and *trait shame* as a covariates, was calculated. The covariates *trait social anxiety*, *F*(1, 108) = 6.56, *p* = .012, $\eta_{p}^{2}$ = .038, and *trait self-compassion* are significantly related to experimental *condition*, *F*(2, 108) = 1.874, *p* = .047, $\eta_{p}^{2}$ = .023, while *trait shame* is not a significant covariate (*p* > .29). Controlling for the effect of *trait social anxiety* and *trait self-compassion*, the significant main effect of *condition* on *shame reduction between t1 and t2* becomes significant, *F*(2, 108) = 3.854, *p* = .024, $\eta_{p}^{2}$ = .045.

## Discussion

The aim of the present study was a) to test an online-adapted method for inducing shame and b) to pilot-test two self-help interventions against heightened shame experiences. The results of this study show that the shame-based *Autobiographical Emotional Memory Task* could successfully induce both shame and fear. Furthermore, the results show that a micro-intervention based on self-compassion can reduce shame significantly better than the control condition although this effect could only be shown for shame and not for fear. An exploratory analysis also showed that trait social anxiety and trait self-compassion moderated this effect. Almost across all measurement time points, more shame and more fear were reported in the high socially anxious group compared to the low socially anxious group.

Confirming the first part of the manipulation check, the *Autobiographical Emotional Memory Task* used in this study was successful in inducing shame and fear and, accordingly, should be further applied in future experimental studies. Contrary to the second part of the manipulation check, shame and fear were induced with a similar intensity, when comparing the effect sizes. Accordingly, this induction procedure cannot be labeled as being shame-specific, which at least in part might be due to fear and shame being overlapping and highly correlated emotional states (Gilbert et al., 1994). However, to further validate the induction procedure, the introduction of a divergent variable, e.g., an emotion such as joy, is clearly recommendable. Furthermore, an induction task which is known to elicit feelings of shame even more precisely would certainly be desirable. In future research, of course, experimenters should further take care for applying the induction with ethical sensibility, as AEMT could lead to increased stress especially in samples with vulnerable individuals.

Even though there is a main effect of social anxiety group, which underlines the link between shame and social phobic symptoms (Fergus et al., 2010; Gilbert & Miles, 2000; Lutwak & Ferrari, 1997), the post-hoc effect did not become significant. Further, the results did not confirm the interaction effect hypothesized in H 2.1, which implies that the induction of shame elicit higher level of shame in subjects with higher compared to lower levels of social anxiety. This might in part be explainable by the non-clinical nature of the sample (with limited variance in social anxiety severity), but it seems more likely that there was a ceiling effect in the socially anxious group, with their initially higher shame experience scoring leaving virtually no room for further increase in shame experiences on the Likert scale. In the future, it might be useful to develop an empirical valence scale for shame (Lishner et al., 2008) that takes such ceiling effects into account.

In addition to exploring induction methods, the focus of the present study was on interventions to change exaggerated shame. Here, in line with H 1, the results show that the micro-intervention based on self-compassion (COMP) reduced shame with a medium effect size and significantly more strongly compared to a control condition (CONT). This finding supports previous ones showing that interventions based on self-compassion can be helpful in regulating shame (Cândea & Szentagotai-Tătar, 2018). This is particularly noteworthy because the control condition was an active distraction task that was also capable of producing an emotion-regulating effect. Moreover, the preliminary results show that trait social anxiety and trait self-compassion moderates this effect, and that these traits could thus influence the effectiveness of self-compassion strategies. However, this would need to be investigated in more detail in future studies. Interestingly, the stronger effect of the COMP condition specifically compared to CONT applies only to shame (and not fear) and thus does not seem to simply reflect a non-specific arousal effect. As the main effect condition became only marginally significant across all three conditions, the results need to be further verified, with the direct comparison between COMP and CONT reaching significance. Future studies should investigate whether any type of active emotion regulation conditions could be similarly effective. The results of the present study however suggest that COMP has benefits compared to active avoidance.

Not confirming to H 1, the comparison with the other active regulation intervention, cognitive reappraisal (REAP), did not show a significantly superior shame reduction of the COMP condition. REAP did not show superiority in shame reduction over the CONT condition either, thereby indirectly indicating that COMP might be preferable in reducing shame. Also contrary to H 2.2, REAP and COMP resulted not in stronger reduction of shame or fear in comparison to the control condition in subjects with higher levels of social anxiety. At the same time, the more socially anxious group reported more shame reduction during the intervention. Thus, this finding supports the proposition that using self-compassion can be a successful approach to regulate shame for individuals with higher social anxiety symptoms (cf. Blackie & Kocovski, 2018; Goldin & Gross, 2010; Koszycki et al., 2016). These results are also promising, in view of the transdiagnostic significance of shame in a number of other psychopathologies e.g. depression (for review: Kim et al., 2011), eating disorders (Nechita et al., 2021), post-traumatic stress disorder (Saraiya & Lopez-Castro, 2016).

### Limitations

The present study has some limitations. Firstly, as this was an online study it could not directly be observed what the subjects did during the experiment and with what level of personal involvement they participated. We tried to experimentally control this limitation from the beginning with a series of open-ended questions. By asking the questions at the end, we hoped that it could reduce the effects of social desirability. In addition, we included subjects in the study conservatively, excluding *n* = 32 subjects from the analyses. Nevertheless, the duration and the demands of the study might have introduced some unknown bias. A second limitation of this study is the non-clinical population, although previous research (Abramowitz et al., 2003) postulated that thoughts and behaviors in psychological disorders differ more in their quantitative rather than qualitative aspects to those observed in non-clinical individuals and that basic aspects of psychological disorders (e.g., emotion regulation) can be investigated on a continuum between non-clinical individuals and patients. Yet, the sample shows on average relatively high social anxiety scores and a relatively wide variation of scores. Both aspects are favourable for investigating our research questions. Thirdly, this was a feasibility study with a piloting character without a formal a priori power analysis, and the study was not preregistered. These aspects are of course inevitable preconditions for possible replication studies in the future. Fourth, the results might have been influenced by responder bias because self-report questionnaires were used for measuring the dependent variable. However, the response tendencies affect all conditions equally and thus should have no influence on differences between the conditions, but rather increase the “noise” of the main effect time. Even though questions concerning shame and fear were not directly posed, further less biased measures, e.g., physiological measures should be included in future studies. Fifth, it cannot be definitively ruled out that the effect is artifactual due to scale effects, even though we controlled for the initial measurement (t0/t1). By scale effects we mean that details of the information are lost due to the small range and the upper and lower limits of the scale. In future studies it would be helpful to use longer-lasting, potentially even more intense interventions and follow-up measurements. Lastly, future experiments might consider recording the broader variable “gender” instead of “sex”.

### Conclusions and Implications for Further Research

The study had two aims: first, to examine the extent to which the *Autobiographical Emotional Memory Task* is a helpful approach to induce shame experimentally in an online setting, and second, to find experimental evidence for superior effects of self-compassion in reducing subclinical shame (when compared with active control conditions). The results show that shame could be effectively induced in an experimental online study with the *Autobiographical Emotional Memory Task,* but that the induction also elicited fear which is why the procedure should be further developed and validated in future studies. In addition, the results show that even a short micro-intervention of self-compassion, unlike cognitive reappraisal, was significantly more efficacious in in down-regulating shame – also in healthy individuals with higher social anxiety symptoms – than a control intervention. Additionally, the traits of social anxiety and self-compassion seem to moderate this effect. These results support the importance of self-compassion in the treatment of shame-related disorders.

## Supplementary Materials

The Supplementary Materials contain the following items (for access see Index of Supplementary Materials below):

- The research data for this study
- The codebook for the dataframe
- The online appendices for the article

Fink-LamotteJ.
HoyerJ.
PlatterP.
StierleC.
ExnerC.
 (2021a). Supplementary materials to "Shame on me? Love me tender! Inducing and reducing shame and fear in social anxiety in an analogous sample"
[Research data]. PsychOpen. https://osf.io/ne48g
10.32872/cpe.7895PMC1086363838356896

Fink-LamotteJ.
HoyerJ.
PlatterP.
StierleC.
ExnerC.
 (2021b). Supplementary materials to "Shame on me? Love me tender! Inducing and reducing shame and fear in social anxiety in an analogous sample"
[Codebook]. PsychOpen. https://osf.io/gkam8
10.32872/cpe.7895PMC1086363838356896

Fink-LamotteJ.
HoyerJ.
PlatterP.
StierleC.
ExnerC.
 (2023). Supplementary materials to "Shame on me? Love me tender! Inducing and reducing shame and fear in social anxiety in an analogous sample"
[Online appendices]. PsychOpen. 10.23668/psycharchives.13165
PMC1086363838356896

## Data Availability

Data and non-copyrighted materials can be publicly accessed (Fink-Lamotte et al., 2021a, 2021b, 2023) or will be made available by the authors on reasonable request.
